# Correlation-based full-waveform shear wave elastography

**DOI:** 10.1088/1361-6560/acc37b

**Published:** 2023-05-18

**Authors:** Abdelrahman M Elmeliegy, Murthy N Guddati

**Affiliations:** 1 North Carolina State University, Raleigh, NC, United States of America; 2 Cairo University, Cairo, Egypt

**Keywords:** wave scattering, inverse modeling, PDE constrained optimization, elastography, ultrasound

## Abstract

Objective. With the ultimate goal of reconstructing 3D elasticity maps from ultrasound particle velocity measurements in a plane, we present in this paper a methodology of inverting for 2D elasticity maps from measurements on a single line. Approach. The inversion approach is based on gradient optimization where the elasticity map is iteratively modified until a good match is obtained between simulated and measured responses. Full-wave simulation is used as the underlying forward model to accurately capture the physics of shear wave propagation and scattering in heterogeneous soft tissue. A key aspect of the proposed inversion approach is a cost functional based on correlation between measured and simulated responses. Main results. We illustrate that the correlation-based functional has better convexity and convergence properties compared to the traditional least-squares functional, and is less sensitive to initial guess, robust against noisy measurements and other errors that are common in ultrasound elastography. Inversion with synthetic data illustrates the effectiveness of the method to characterize homogeneous inclusions as well as elasticity map of the entire region of interest. Significance. The proposed ideas lead to a new framework for shear wave elastography that shows promise in obtaining accurate maps of shear modulus using shear wave elastography data obtained from standard clinical scanners.

## 1. Introduction

Soft tissue stiffness is an important biomarker in the diagnosis of cancerous tumors (Boyd *et al* 2001, Mccormack and Dos Santos Silva 2006). It is known from the pathology viewpoint that dense breast tissue is one of the most important factors for developing breast carcinoma (Provenzano *et al* 2008). Magnetic Resonance Elastography (MRE, (Mariappan *et al* 2010)), and ultrasound elastography (Sigrist *et al* 2017) are among the most advanced modalities that are used to provide a map of soft tissue stiffness (elasticity map, hence the name, elastography). Although MRE has been quite successful in this regard, ultrasound elastography started to gain more attention over the last decade owing to its shorter acquisition time, lower cost, portability, and safety (Guasch *et al* 2020). We refer the reader to (Sarvazyan *et al* 2011) for a more comprehensive review of the elastography imaging techniques.

Generally, there are two main elastography techniques, static and dynamic (Gennisson *et al* 2013, Shiina *et al* 2015, Sigrist *et al* 2017). In static elastography, Hook’s law-based inversion is used to estimate the elastic modulus from the measured displacement field. However, because the boundary conditions between different tissues are not precisely known, these techniques cannot quantitatively assess the mechanical properties of soft tissues (Barbone and Bamber 2002). On the other hand, dynamic elastography, e.g. shear wave elastography (SWE), can provide a quantitative elasticity map with better contrast, by exciting the tissue with a shear wave that is generated by focusing acoustic beams inside the tissue (the resulting push is referred to as acoustic radiation force (ARF), ARF (Sigrist *et al* 2017)). These shear waves are then tracked by an ultrafast ultrasound scanner which tracks the wavefield over the entire region of interest (ROI) with high accuracy (Deng *et al* 2017). For a more comprehensive review of the current ultrasound SWE techniques and their advantages, one can refer to (Berg *et al* 2012, Taljanovic *et al* 2017).

Over the last decade, several dynamic reconstruction techniques have been developed which can be classified into two main categories, local and global (Arnal *et al* 2013). Some examples of local reconstruction techniques are, direct inversion of wave equation (Sandrin *et al* 2002, Nightingale *et al* 2003, Catheline *et al* 2004, Lin and McLaughlin 2009), Time of Flight (ToF (Palmeri *et al* 2008); Tanter *et al* 2008, Song *et al* 2012, 2014, Carrascal *et al* 2017), Plane Wave Elastography (PWE (Khodayi-mehr *et al* 2021)), and phase velocity based inversion (Chen *et al* 2004, 2009, Budelli *et al* 2016, van Sloun *et al* 2017, Kijanka and Urban 2019). Direct wave equation inversion is straightforward but requires the wavefield to be known in the entire ROI. Furthermore, direct inversion of wave equation results in an operator that is highly sensitive to noise. These drawbacks make direct inversion not a reliable imaging technique (Deffieux *et al* 2012). On the other hand, ToF, PWE, and phase velocity-based methods assume that waves propagate with plane wavefronts. In addition, the medium is assumed to be locally homogeneous and have no scattered waves (Deffieux *et al* 2011). For instance, ToF constructs a map of the shear wave speed after calculating the time delays for all spatial points in the direction of the wave propagation (Tanter *et al* 2008, Song *et al* 2014). This method is most commonly used in commercial scanners in different contexts, e.g. Fast Shear Compounding (Song *et al* 2014), Supersonic Shear Imaging (Athanasiou *et al* 2010), and comb-push ultrasound shear elastography (Denis *et al* 2015).

Global (dynamic) reconstruction techniques (Doyley 2012), e.g. full-waveform inversion (FWI), alleviate the disadvantages of the local reconstruction techniques by more accurately capturing the wave propagation inside the tissue, i.e. no assumption of wave propagation is made and all types of wave phenomena, including refractions, reflections and scattering associated with heterogeneities are accurately captured. Another attractive feature of the global reconstruction approaches is that noise can be readily handled (Arnal *et al* 2013, Ghosh *et al* 2017, Ghavami *et al* 2020). However, FWI is computationally expensive, and, more importantly, it is highly nonlinear potentially leading to erroneous reconstructions. Moreover, the push amplitude is not precisely known, which may result in failure of accurate reconstruction. Other global techniques such as semi-analytical approaches (Hadj-Henni *et al* 2008, Montagnon *et al* 2013, 2014, Bernard and Cloutier 2017) provide good results with less computational cost, but are limited to simple geometries and cannot provide elasticity maps with arbitrary heterogeneities.

The choice of the cost functional and the parameterization are two main factors that affect the robustness of any reconstruction algorithm. In contrast to the conventional least-squares ($L_{2}$) cost functional, the cross-correlation (CC) based cost functional emphasizes the phase information by measuring the zero-lag cross-correlation between the measured and the simulated wavefields, rendering the inversion insensitive to the push amplitude. In addition, CC functionals are less nonlinear, hence less sensitive to the initial guess. Several studies in the geophysics field have demonstrated the success of the CC functionals (Van Leeuwen *et al* 2010, Choi and Alkhalifah 2012, Liu *et al* 2016, Tao *et al* 2017, Wu and Alkhalifah 2018). Although there is plenty of research that considers the CC functionals in geophysics, to the best of our knowledge, we are not aware of any study that proposes the CC cost functionals for the ultrasound SWE.

In light of the above discussion, in this work, we address the issues associated with the FWI for ultrasound elastography and propose a CC cost functional, that leads to reducing the nonlinearity of the FWI problem and results in a reconstruction algorithm that is independent of push amplitudes, less sensitive to the initial guess, and has a better convergence behavior compared to the classical $L_{2}$ cost functional. Furthermore, we note that conventional ultrasound transducer measures a component of the wavefield in a single plane but the objective is to map a 3D tumor. Motivated by this, we focus on the reconstruction of 2D elastography images from measurements on a single line. Building on this proof-of-concept investigation, our eventual goal is to develop 3D imaging of tumors using ultrasound measurements using existing ultrasound transducers.

The rest of the paper is organized as follows. Section 2 presents the details of proposed reconstruction algorithm based on CC cost functional. Section 3 presents a comprehensive study of the proposed approach using synthetic 2D experiments, where errors in the reconstruction are examined as a function of common input errors in ultrasound elastography. We then discuss the outcome of the work as well as potential future investigations in section 4, followed by concluding remarks in section 5.

## 2. Methods

The basic approach of our reconstruction method is to iteratively modify the elasticity map until the simulated particle velocities match well with the measured particle velocities. Two key aspects of this process are (a) the forward model, which provides the simulated responses for any given estimates of elastic properties, and (b) the cost functional, which encapsulates the goodness of the match between measured and simulated responses. In this section, we first review the forward model, which is essentially governed by 2D wave equation. In later subsections, we turn our attention to the cost functional, first reviewing the common least-squares approach, followed by the presentation of the proposed CC cost functional. Also presented is the summary of the gradient optimization algorithm utilized in our study, along with the derivation of appropriate gradients that are central to the algorithm. Finally, we describe the overall setup for the *in-silico* experiments conducted in section 3.

### 2.1. Forward model

In this work, we consider $\Omega_{\text{ROI}}\in {\mathbb{R}}^{n}$ to denote the ROI in soft tissues. The methodology is general and is expected to extend to 3D as well, but we focus on 2D problems in this study, i.e. n=2 in the present context. Further, we assume that the medium is isotropic and linearly elastic (and ignore any viscous effect at this time). The assumption of linearity follows the work in (Palmeri *et al* 2017) that ARF is very low in amplitude that the nonlinear effects do not enter into picture. Moreover, we consider the elastic modulus to not vary within the observation time, e.g. we ignore elastic modulus changes due to interstitial fluid pressure changes associated with poroelasticity. Finally, we focus on the scenario with ARF is applied inside the ROI, generating shear waves that travel through the ROI. Given these assumptions, we can represent the shear wave propagation inside the ROI using the scalar wave equation:

(1) $$-\nabla \cdot \left(\mu \left(x\right)\nabla u\left(x,t\right)\right)+\rho \ddot{u}\left(x,t\right)=f\left(x,t\right),x\in \Omega_{\text{ROI}},$$

where u(x,t) is the wavefield, μ(x) is the shear modulus of the tissue, ρ is the tissue density and f(x,t) is the ARF push function. Further simplification can be applied by noticing that

(2) $$c\left(x\right)=\sqrt{\frac{\mu \left(x\right)}{\rho }},$$

where c(x) is the shear wave speed inside the soft tissue. This results in

(3) $$-\nabla \cdot \left(c^{2}\nabla u\right)+\ddot{u}=f,$$

where explicitly showing the dependency on time and space is omitted for convenience. Here, we chose to solve equation (3) in the frequency domain so that we have better control on the numerical dispersion especially at higher frequencies, and to facilitate parallelization over frequencies, leading to a more computationally efficient reconstruction. Thus, we Fourier transform equation (3) in time, resulting in the reduced wave equation in frequency (ω) domain

(4) $$-\nabla \cdot \left(c^{2}\nabla 𝒰\right)-\omega^{2}𝒰=ℱ,$$

where 𝒰 and ℱ are respectively the wavefield and the forcing function in (x, ω) space (Fourier transforms of u and f). It is well known that most of the soft tissue are viscous, thus the wave damps out as it moves away from the ARF. Also, we are assuming that the tumor is small relative to the organ and it exists relatively far from the surface. Given these observations, we assume that the background medium is homogenous around the tumor and the waves travel outside the ROI without being reflected back into the ROI. Therefore, we utilize the radiation condition on the boundary. The boundary value problem can be stated as follows: given c,ω and ℱ, find 𝒰 such that

(5) $$-\nabla \cdot \left(c^{2}\nabla 𝒰\right)-\omega^{2}𝒰=ℱ,x\in \Omega_{\text{ROI}}\in {\mathbb{R}}^{2},\;\nabla 𝒰\cdot n=\Lambda 𝒰,x\in \Gamma \equiv \partial \Omega ,$$

where n is the outward normal and Λ is the Dirichlet-to-Neumann operator representing the radiation boundary condition, often approximated by an absorbing boundary condition (Guddati and Lim 2006).

Equation (5) is typically solved using a discretization based method such as finite element or finite difference methods. The discretized version of the scalar wave equation (5) can then be written as

(6) 𝓐u=b,

where 𝓐 is the discrete wave operator obtained using e.g. finite element or finite difference methods, u and b are respectively the nodal displacements (wavefield) and force vectors (ARF push). In this work, we utilize the standard finite element method with 2D bilinear finite elements (4-node bilinear element) for modeling the physical ROI described in equation (5), leading to finite $\left(N_{\text{dofs}}\right)$ degree of freedoms (DOFs). In addition, the absorbing boundary condition in equation (5) is modeled using perfectly matched discrete layers (Guddati and Lim 2006) which is a discrete version of the well-known perfectly matched layers (Berenger 1994). Typically, 3–5 PMDL layers are sufficient to absorb the waves efficiently, leading to an additional $m_{\text{pmdl }}$ DOFs. This results in a linear system of equations represented by equation (6), where the size of matrix 𝓐 is $\left(N_{\text{dofs }}+m_{\text{pmdl}}\right)\times (N_{\text{dofs}}+\;m_{\text{pmdl}})$, and the size of vectors u and b is $\left(N_{\text{dofs}}+m_{\text{pmdl}}\right)$.

### 2.2. Conventional reconstruction approaches

In this section, we review the widely used reconstruction method based on the adjoint-based gradient optimization, which minimizes the misfit between the measured and predicted responses in a least-squares sense. The objective of the reconstruction is to obtain the distribution of the shear wave speed in the ROI. The discretized version of the shear wave speed distribution takes the vector form, which is the model parameter vector (m) of size m (m is related to the spatial resolution of the reconstruction; higher the resolution, bigger the value of m). The goal of reconstruction is to find the optimizing model parameter vector $\overset{ˆ}{m}$, i.e. optimal shear wave velocity distribution, by minimizing a cost functional 𝒥(m) that captures the misfit between the measured and predicted responses:

(7) $$\overset{ˆ}{m}=\text{argmin }𝒥\left(m\right).$$

Conventional choice of the cost functional is related to least-squares minimization of the misfit, which is described below, along with its gradient, which is an important quantity used in many optimization methods.

#### 2.2.1. Least-squares cost functional and its gradient

Let d in $\Omega_{\text{data}}\subseteq \Omega_{\text{ROI}}$ and u(m) be the measured and the predicted wavefields, respectively. The least-squares cost functional is defined as

(8) $$𝒥\left(m\right)=\frac{1}{2}{\left(𝓟u\left(m\right)-d\right)}^{†}(𝓟u\left(m\right)-d),$$

where 𝓟 is an observation operator, which is a projection matrix that restricts the full wavefield u to the measurement locations, and † represents conjugate transpose.

The goal of the reconstruction is to find the minimizer of the cost functional. To this end, we follow the standard gradient optimization approach; we summarize the basic idea here and the reader is referred to (Tarantola 1984) for further details: We start with Taylor series expansion of equation (8) in the vicinity of a starting point $m_{0}$:

(9) $$𝒥\left(m_{0}+\Delta m\right)\approx 𝒥\left(m_{0}\right)+\Delta m^{T}g+\frac{1}{2}\Delta m^{T}𝓗\Delta m,$$

where g is the gradient vector, 𝓗 is the Hessian matrix and T represents matrix transposition. Assuming that the Hessian is invertible, the estimate of the perturbation Δm which minimizes the functional in equation (9) is determined by

(10) $$\Delta m=-{𝓗}^{-1}g.$$

For a small number of model parameters m, the finite difference approximation could be utilized for the gradient computation (by perturbing each parameter). However, when m is large, taking finite differences by perturbing each parameter will be highly inefficient. Instead, we utilize the adjoint formulation for the gradient calculation where the ${\text{i}}^{\text{th }}$ component of the gradient g can be expressed as

(11) $$g_{i}=\frac{\partial 𝒥\left(m\right)}{\partial m_{i}}=ℜ\left\{u^{†}\frac{\partial {𝓐}^{†}}{\partial m_{i}}v\right\},i=1,\ldots ,m,$$

where v is the adjoint variable, which can be obtained by solving the adjoint equation:

(12) $${𝓐}^{†}v={𝓟}^{T}\left(d-𝓟u\left(m\right)\right)$$

where d−Pu(m) is the misfit computed at the measurement’s location, and ${𝓟}^{T}$ is an expansion operator that has one as entries corresponds to the measurements and zeros otherwise. ${\left(𝓐\right)}^{-1}{𝓟}^{T}\left(d-Pu\left(m\right)\right)$ can be interpreted as a backpropagation of the misfit to the entire computational domain (see e.g. (Pratt *et al* 1998).).

Gradient-based reconstruction requires an initial model to start the iterations. If the initial model is significantly different from the ground truth, the optimization may converge to an incorrect solution, since the $L_{2}$ cost functional tends to have many local minima (example is provided in section 3.1). The situation with imaging becomes worse at higher frequencies due to the phenomena of cycle skipping (Wu and Alkhalifah 2018). In addition, as noted earlier, if the push amplitude is not precisely known, the iterative minimization may lead to a local minimum which may be far away from the global minimum, leading to erroneous reconstruction, thus the need for a method that can mitigate all or some of these challenges.

### 2.3. Proposed reconstruction methodology

In this section, we present the cross-correlation (CC) based functional as an alternative to the classical $L_{2}$-norm of the misfit. Such functionals have been used in geophysical inversion with better success compared to the $L_{2}$-norm of the misfit. Essentially, CC functional captures the correlation between the measured and the predicted wavefields by computing the cross-correlation of the measured and the predicted wavefields. Hence, the cost functional is independent of the push amplitudes which is often not precisely known. More importantly, CC functionals emphasize the phase information as opposed to amplitude mismatch leading to a significantly better behaving algorithm with no additional cost. In what follows, we present the CC functional and derive its gradient based on the adjoint formulation.

#### 2.3.1. Cross-correlation (CC) cost functional

We propose to use a CC cost functional 𝒥(m) that measures the (zero-lag) correlation between the two signals d and u(m) can be defined as follows

(13) $$𝒥\left(m\right)=1-\frac{|d\cdot \left(𝓟u\left(m\right)\right){|}^{2}}{{\left(∥d∥\;∥𝓟u\left(m\right)∥\right)}^{2}}=1-\frac{{\left(𝓟u\left(m\right)\right)}^{†}d\;\;d^{†}\left(𝓟u\left(m\right)\right)}{d^{†}d\;{\left(𝓟u\left(m\right)\right)}^{†}\left(𝓟u\left(m\right)\right)}.$$

The second term is essentially the square of the normalized cross-correlation. The term is negated to convert the problem to a minimization problem. Note that squaring of cross-correlation is introduced as it appears to increase the convexity of the cost functional. It also facilitates cleaner derivation of the gradient, which is presented below.

#### 2.3.2. Gradient of CCfunctional

Using the adjoint formulation, the gradient can be written as

(14) $$g_{i}=ℜ\left\{\alpha \left(u^{†}\frac{\partial {𝓐}^{†}}{\partial m_{i}}v\right)+{\left(\alpha \left(u^{†}\frac{\partial {𝓐}^{†}}{\partial m_{i}}v\right)\right)}^{†}-\beta \left(u^{†}\frac{\partial {𝓐}^{†}}{\partial m_{i}}\ell +{\left(u^{†}\frac{\partial {𝓐}^{†}}{\partial m_{i}}\ell \right)}^{†}\right)\right\},$$

which can be simplified as

(15) $$g_{i}=2ℜ\left\{\alpha \left(u^{†}\frac{\partial {𝓐}^{†}}{\partial m_{i}}v\right)-\beta \left(u^{†}\frac{\partial {𝓐}^{†}}{\partial m_{i}}\ell \right)\right\},\;\alpha =\frac{1-𝒥\left(m\right)}{{\left(𝓟u\left(m\right)\right)}^{†}d},\beta =\frac{1-𝒥\left(m\right)}{{\left(𝓟u\left(m\right)\right)}^{†}\left(𝓟u\left(m\right)\right)},\;v={\left({𝓐}^{†}\right)}^{-1}{𝓟}^{T}d,\ell ={\left({𝓐}^{†}\right)}^{-1}{𝓟}^{T}𝓟u\left(m\right).$$

This can be further simplified as

(16) $$g_{i}=2ℜ\left\{u^{†}\frac{\partial {𝓐}^{†}}{\partial m_{i}}\left(\alpha v-\beta \ell \right)\right\}=2ℜ\left\{u^{†}\frac{\partial {𝓐}^{†}}{\partial m_{i}}\overset{˜}{v}\right\},$$

(17) $$\overset{˜}{v}={\left({𝓐}^{†}\right)}^{-1}{𝓟}^{T}\left(\alpha d-\beta 𝓟u\left(m\right)\right)$$

Comparing the adjoint in equations (17) and (12), the right-hand side in equation (12) (misfit) is modified by introducing α and β. Intuitively, α and β can be interpreted as shifting and weighing factors; when α=β=0.5, we get back equation (12). Essentially comparing equations (17) and (12) indicates that the computational cost of adjoint, and thus gradient computation is essentially identical between traditional least-squares and proposed CC functional.

#### 2.3.3. Reconstruction algorithm

Given that the cost functional is defined and the associated gradients are derived, a standard gradient-based optimization framework can be utilized to perform the reconstruction. Based on our prior experience with seismic imaging problems (Eslaminia *et al* 2022), we utilize BFGS algorithm (Broyden 1970, Fletcher 1970, Goldfarb 1970, Shanno 1970), which turned out to perform well for the numerical examples explored in this work. There are other gradient-based optimization algorithms that could be used in replace of BFGS method (Nocedal and Wright 2006), but BFGS method is well-known to be an effective choice for material inversion (see e.g. (Doyley 2012).) and we have not explored other methods given the success we had with the BFGS method for the current work. The steps are presented in Algorithm 1.

**Algorithm 1.** Proposed reconstruction algorithm based on CC cost functional.

Given the measurements d. Discretize the ROI into small pixels representing the shear modulus spatial distribution $m_{0}$. Given an initial model $m_{0}$ and stopping criterion e.g. ∥g∥⩽ε, minimize the cost functional in equation (13) over all frequencies and pushes until convergence. Each iteration k of the minimization process involves the following steps: Obtain the preliminary step $\Delta m_{k}$ in equation (10) using the following steps: Compute the modified back propagated misfit $\tilde{v}$ in equation (17). This requires one adjoint solve. Compute the gradient $g_{k}$ using equation (16). Solve equation (10) using the BFGS algorithm to get the step $\Delta m_{k}$. Using cubic line search, minimize the cost function in equation (13) to compute the step size α. Update the model: ${\overset{ˆ}{m}}_{k}=m_{k}+\alpha_{k}\Delta m_{k}$. Repeat step 1 until $∥g{∥}_{k}<\varepsilon$.

#### 2.3.4. In silico experimental setup

**Figure 1.**
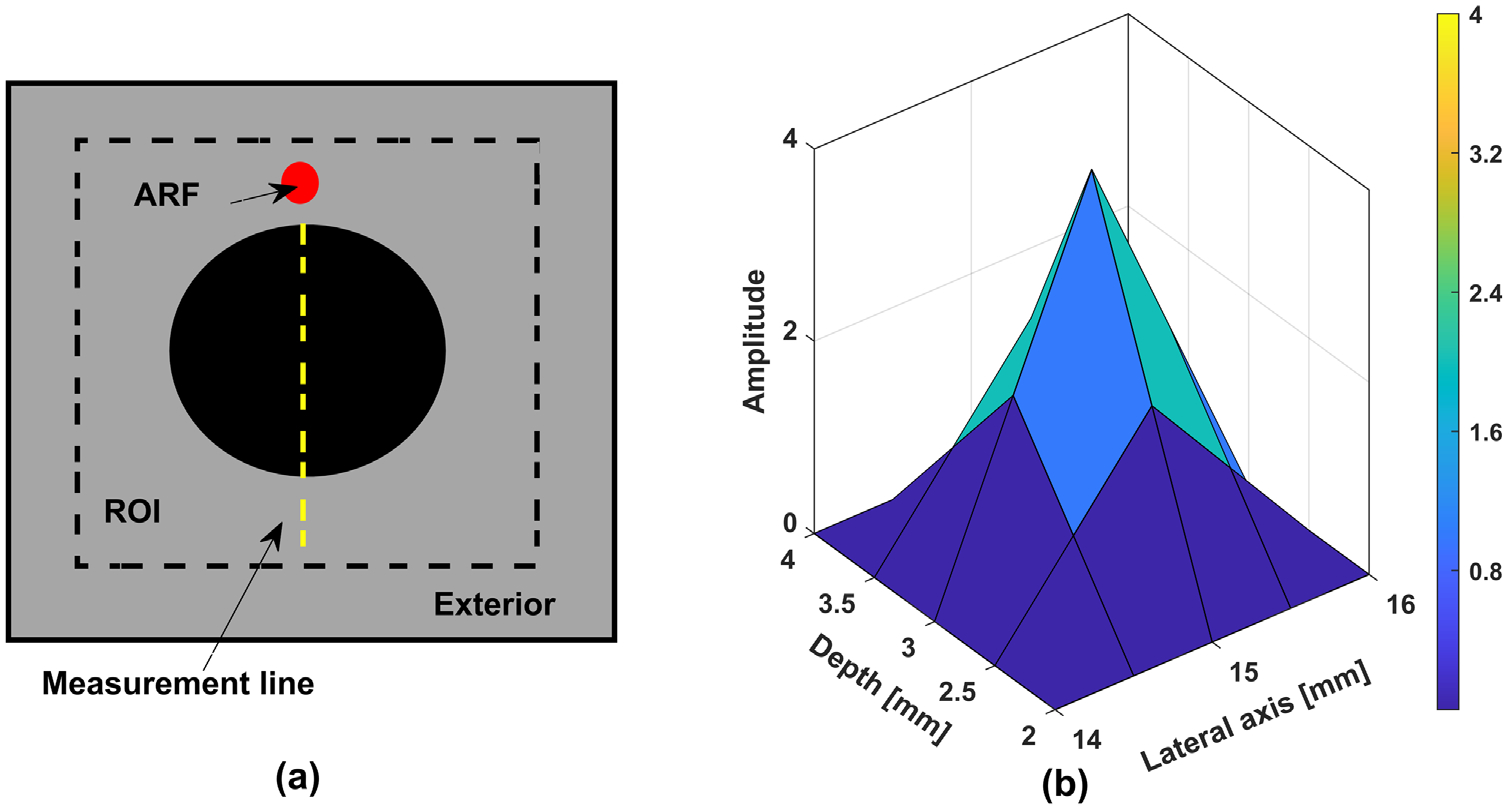
(a) The default setup, and (b) the default ARF configuration.

We start with the observation that conventional ultrasound scanners measure (single component of) the particle velocity in a single plane, whereas the tumors to be imaged are 3D in nature. We encapsulate this reality in a simpler setting, where our goal is to obtain a 2D image given the measurement on a single line (see figure 1(a)). The expectation is that if a method is effective in the proposed 2D setting, it could be extended to realistic imaging of 3D tumors using SWE data. In section 3, we present results using *in silico* experiments to illustrate the performance of the proposed method in two different scenarios of SWE, first in an idealized setting and then in more realistic settings. In this section however, we describe the overall setup for the *in silico* experiments conducted in section 3, borrowing from past work (Bernard and Cloutier 2017, Ghosh *et al* 2017, Khodayi-mehr *et al* 2021); any deviations from this general setup are mentioned in each of the specific experiments.

**Figure 2.**
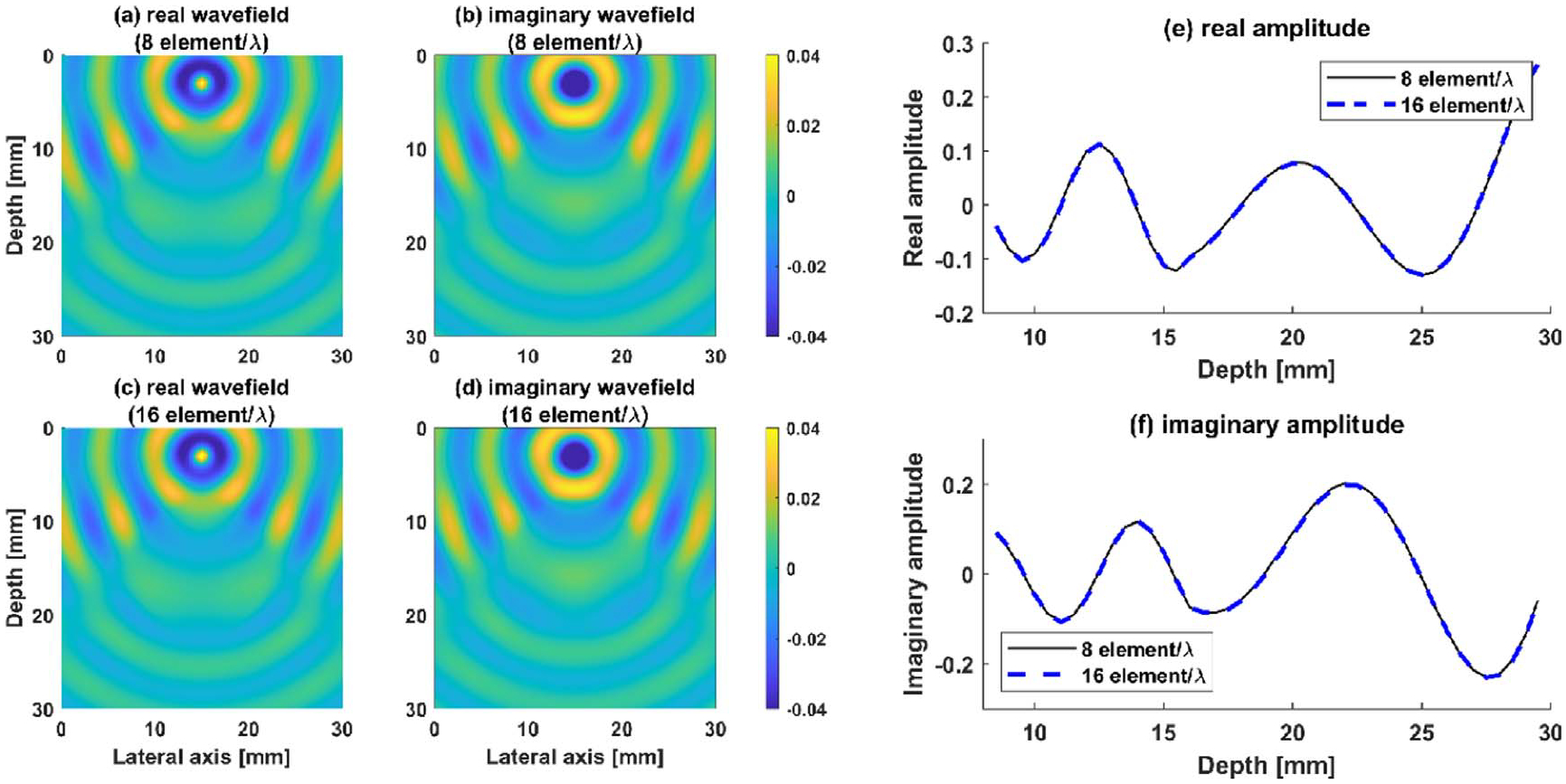
Comparison of normalized wavefields at 500 Hz for different mesh resolutions: (a), (b) real and imaginary parts of responses using 8 elements per wavelength, (c), (d) real and imaginary part using 16 elements per wavelength, (e), (f) comparison of real and imaginary parts of the wavefield sampled on the vertical line at the middle of the ROI.

The region of interest (ROI): The default ROI is taken as 30 mm×30 mm square. The inclusion shape can vary but its nominal diameter is 14 mm. The shear wave velocity of the background and the inclusion are 2.88 m ${\text{s}}^{-1}$ and $5.16{\text{ m s}}^{-1}$ respectively. As mentioned earlier in section 2.1, we solve the forward model in frequency domain using the finite element method. Following a 60×60 finite elements mesh, which guarantees at least eight elements per wavelength. This is consistent with the widely used protocol for wave simulations (see e.g. (Marburg 2008). and figure 2 for an illustration of the resulting accuracy).

Acoustic radiation force (ARF): In *in vivo* settings, the problem including the ARF is 3D in nature and can be accurately modeled with the help of acoustic simulation (Palmeri *et al* 2017). Since our idealization is in 2D, we approximate the ARF push to be (discretized) sine squared pulse in space. Unless otherwise mentioned, we use the default in figure 1(b) when generating the synthetic and predicted wavefields. In section 3.2.4, we demonstrate that the proposed method is not sensitive to the ARF push Configuration.

##### Choice of frequency content:

In this study, we consider different frequency contents depending on the complexity of the reconstruction. The frequency content is chosen so that there is a wide range of wavelengths contributing to the reconstruction, thus leading to a proper spatial resolution to image the tumor (as the method is to be applied to heterogeneous inclusions, as illustrated in section 3.4). For instance, the lowest frequency is chosen so that the longest wavelength is contained in the ROI. On the other hand, the maximum frequency is chosen so that it can resolve the smallest object to be imaged (better resolution). Similarly, the frequency increment is as big as 100 Hz when reconstructing handful parameters as in section 3.1, and as small as 10 Hz when reconstructing hundreds of parameters as in section 3.4.

##### Inversion parameters:

We start with assuming that the inclusion is homogenous, and the background modulus is known, and only reconstruct inclusion shear wave speed as in section 3.2 (one parameter). Then, in section 3.3, we assume that the background modulus is also unknown but homogeneous and reconstruct both the shear wave speeds of the background and inclusion (two parameters). Finally, in section 3.4, we present the most complicated scenario where we assume that the entire ROI is heterogenous and reconstruct the shear wave speed distribution (several hundreds of parameters).

## 3. Results

### 3.1. Preliminary comparison with least-squares functional

Before presenting the results using realistic *in silico* SWE experiments, we illustrate the main differences between the performance with $L_{2}$ and CC functionals using a hypothetical experiment, where a horizontally propagating plane (shear) wave is scattered by an inclusion of diameter 20 mm, which has an analytical solution as given in (Eringen and Şuhubi 1978). The shear moduli of the background and the inclusion are 12 and 36 kPa respectively. We consider the measurement strictly inside the inclusion, on the horizontal diameter sampled every 1 mm, leading to a total of 18 data points. The initial estimate of the inclusion modulus is assumed to be the same as the background modulus of 12 kPa.

**Figure 3.**
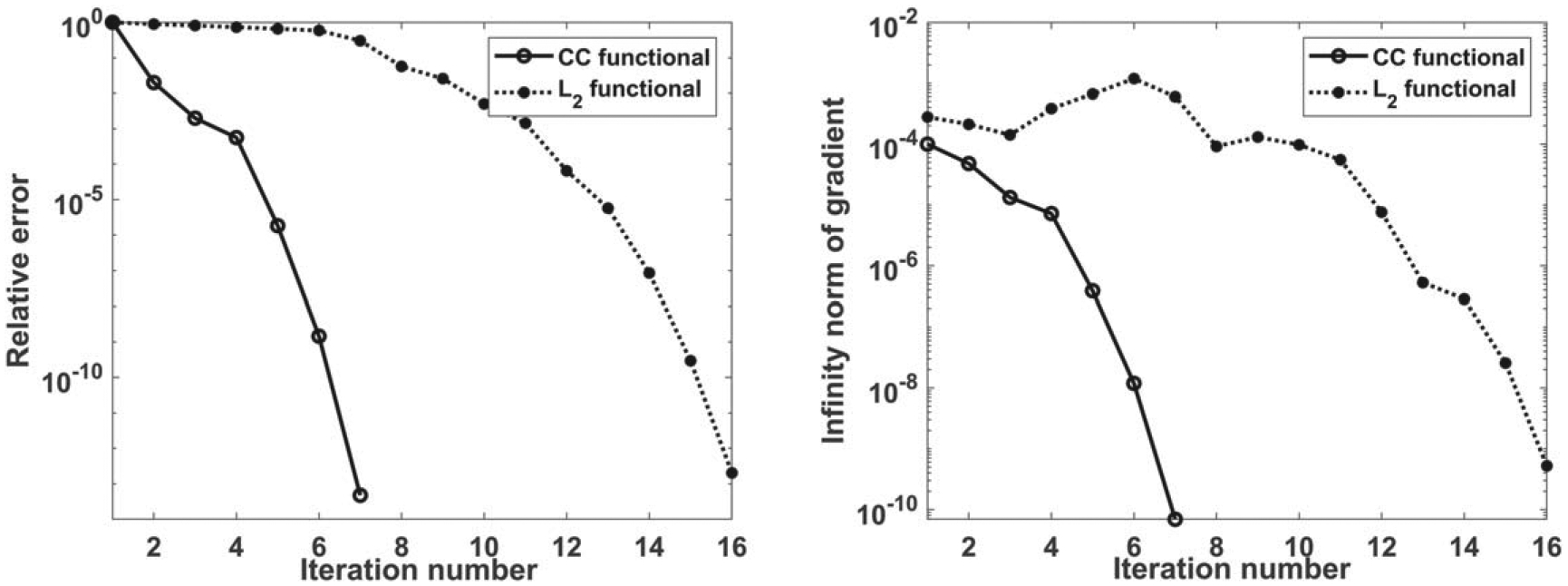
Convergence behavior, relative error (left) and gradient (right).

**Figure 4.**
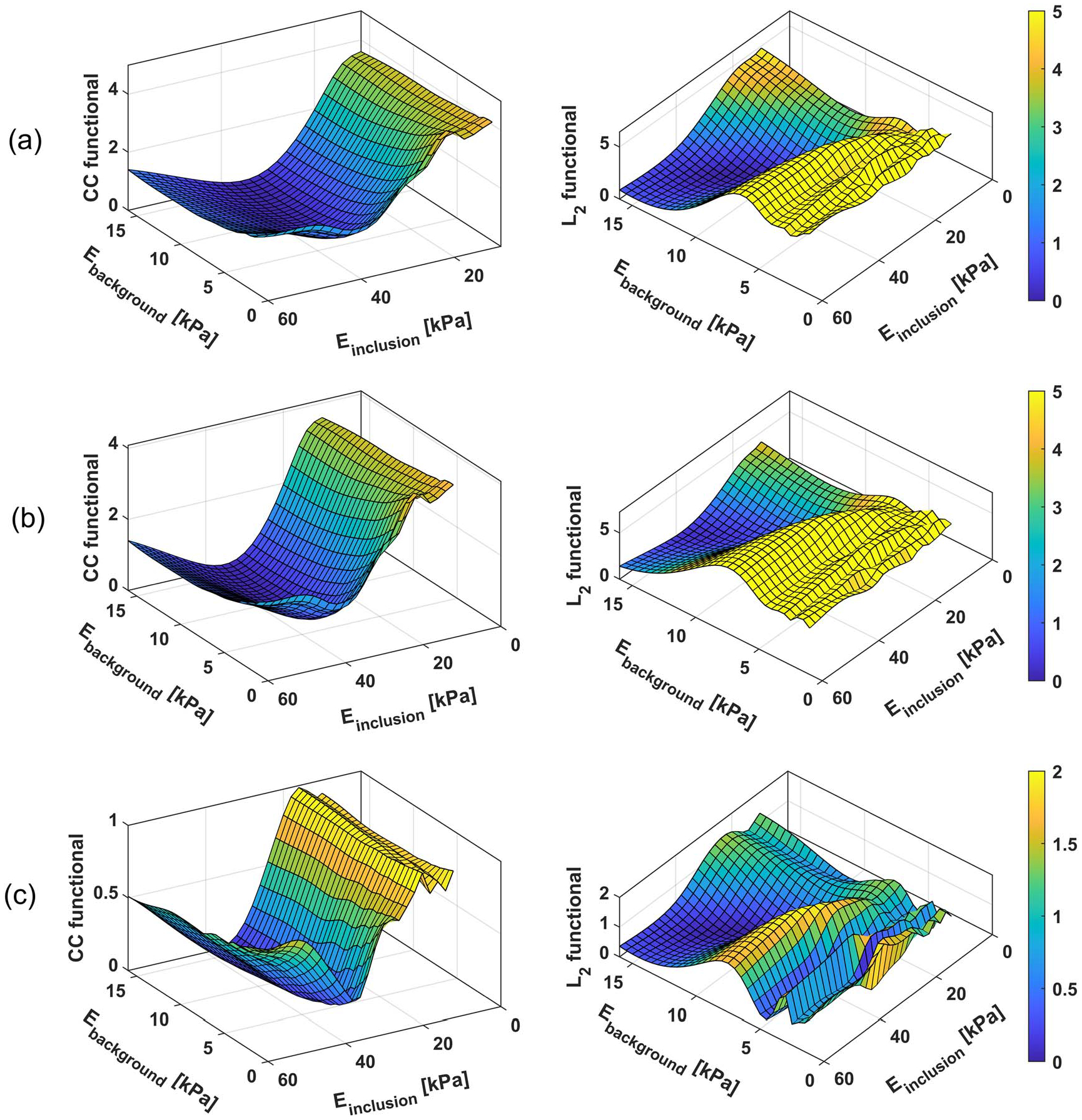
Comparison between $L_{2}$ functional and the proposed correlation functional (a) considering noise-free solution and using the entire frequency range, (b) after introducing noise, (c) noise-free solution limited to a single frequency of 500 Hz.

The convergence behavior of $L_{2}$ and CC cost functionals are presented in figure 3. In addition, the shapes of the cost functions are shown in figure 4(a) (this is possible because the current example involves two parameters). Clearly, the terrain of the CC functional is less complex and more convex than that of the $L_{2}$. This translates to faster convergence leading to increased efficiency and robustness when CC functional is utilized.

#### Effect of push amplitude error.

To test the reconstructability using the two functionals when the amplitude of the incident wave is not precisely known, we intentionally introduced error in the amplitude of the incident wave. Figure 4(b) shows the shapes of the two functionals with erroneous push amplitude. As expected, the CC functional has not been altered, indicating that the global minimum remains at the correct location (background modulus = 12 kPa, inclusion modulus = 36 kPa). On the other hand, the global minimum for $L_{2}$ functional is naturally altered, which corresponds to the incorrect (background modulus = 14 kPa, inclusion modulus = 35.1 kPa). As expected, the reconstruction using Algorithm 1 is successful in estimating the true values of both the background and inclusion when using CC functional, whereas it fails when conventional $L_{2}$ functional is used.

#### Behavior at higher frequencies.

As discussed earlier, FWI is highly nonlinear problem, thus requiring a good initial estimate of the model parameter. Bad initial estimate would result in optimization being trapped in one of the many local minimums. This problem worsens at higher frequencies as seen in figure 4(c), which shows both functionals computed at 500 Hz. Comparing figures 4(c) and (a), clearly, we can see the nonlinearity of the $L_{2}$ functional computed at 500 Hz in figure 4(c) is significantly higher compared to that for the entire frequency range (i.e. 100–500 Hz) shown in figure 4(a); this can be attributed to the phenomenon of cycle-skipping (Van Leeuwen *et al* 2010, Choi and Alkhalifah 2012, Liu *et al* 2016, Tao *et al* 2017, Wu and Alkhalifah 2018). The worsening nonlinearity, while also exists, is significantly less for the CC functional. Thus, unlike the case of $L_{2}$ functional, the problem of converging to a local minimum does not appear to be an issue for CC functional.

#### Effect of initial guess.

Examining figure 4, it is clear that the basins of the global minima are wider for CC functional than $L_{2}$ functional. This indicates that CC functional will have a better chance of convergence to the true values and not get trapped at a local minimum, even when the initial guess is not close to the ground truth.

### 3.2. Homogeneous inclusion with known background modulus

In this section, we consider the estimation of the inclusion modulus (equivalently, shear wave speed), assuming that the background shear wave speed is known from other modalities (e.g. localized SWE (Palmeri *et al* 2008, Tanter *et al* 2008, Song *et al* 2012, 2014, Carrascal *et al* 2017, Khodayi-mehr *et al* 2021) that relies on the local homogeneity assumption, which is often valid for the background). In particular, we examine the robustness of the reconstruction process with respect to the complexity of inclusion shape, noise that is expected in real data, error in push amplitude and signature, as well as errors in the shape of the inclusion. The default parameters of the *in silico* experiments described in the remainder of section 3 are already discussed in section 2.3.4.

#### 3.2.1. Effect of the complexity of the inclusion shape

**Figure 5.**
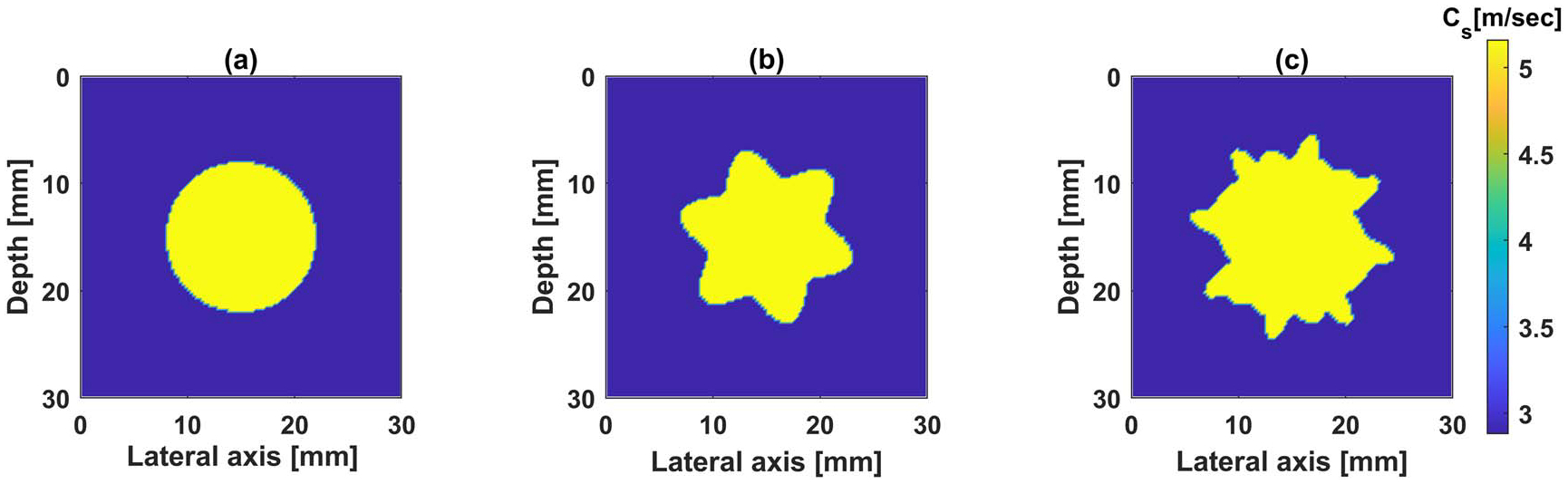
Different inclusion shapes considered in this study (a) circle, (b) star, and (c) irregular.

Three different inclusion shapes are considered, circular, star, and irregular, as shown in figure 5. We use a single frequency of 500 Hz for the reconstruction, and 30 synthetically generated measurements on a single line are used for the reconstruction. The initial estimate of the inclusion shear wave velocity is assumed to be the same as that of the background, i.e. $2.88{\text{ m s}}^{-1}$.

**Figure 6.**
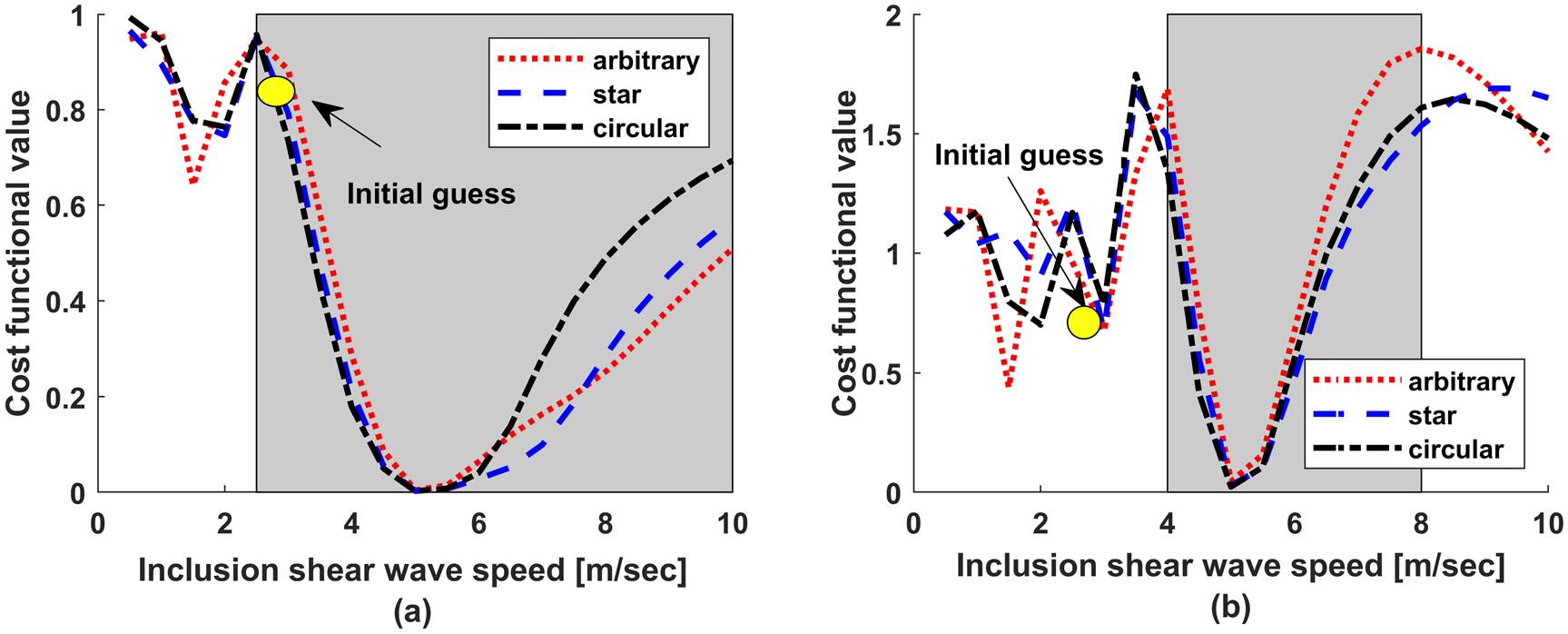
Nonlinearity of the cost functional for the three different inclusion shapes when using CC functional (a) and $L_{2}$ functional (b). The grey area indicates the approximate region of expected convergence. The yellow circle indicates the initial guess.

For all the shapes, CC functional accurately estimates the inclusion shear wave speed, whereas $L_{2}$ functional fails in reconstructing the true shear wave speed of the inclusion. This is explained by observing the cost functional plots in figure 6: given the initial guess (yellow circle), the algorithm with CC functional (figure 6(a)) will converge to the correct solution, whereas it will get trapped at the nearest local minimum when the $L_{2}$-functional (figure 6(b)) is used. Also, by examining figure 6, we see that the CC functional behaves better that the $L_{2}$ functional even for the star shapes inclusion. Given this and the observations in the previous section, we limit the future synthetic experiments to CC functional, which is the main focus of this work.

#### 3.2.2. Effect of noise

Noisy measurement affects the quality of the reconstructed images. Here, we test the proposed approach with noisy measurements (synthetic data). To mimic the measurement noise in real data, the synthetic data is polluted with additive Gaussian white noise, with three different signal-to-noise ratios (SNR) of 30, 20, and 10 decibels (dB). We utilize the example of irregular inclusion shown in figure 5(c), with the same input parameters. Initial estimate of the velocity is taken as the same as the background wave velocity, i.e. $2.88{\text{ m s}}^{-1}$. We fix the shape and invert for the shear wave speed in the inclusion. Three realizations per each SNR level are inverted and we summarize the average reconstructed values in table 1. For all the SNR levels, the proposed framework is able to reconstruct the shear wave speed in the inclusion with errors less than 0.5%.

#### 3.2.3. Effect of error in the assumed inclusion shape

In some situations, the shape of the tumor might not be precisely known. In this case, an approximate shape from a B-mode image may be utilized to start the inversion. Motivated by such scenario, we utilize the irregular inclusion in figure 5(c) to synthetically construct the measured wavefield (with 10 dB noise), then use a circle with a diameter of 14 mm to approximate the true shape and perform the reconstruction. The true shear wave speed of the inclusion is the same as in the previous experiments (i.e. $5.16{\text{ m s}}^{-1}$). The reconstructed shear wave speed is $5.49{\text{ m s}}^{-1}$ which corresponds to a relative error of 6.5%, indicating that the proposed framework is not overly sensitive to error in inclusion shape.

#### 3.2.4. Effect of errors in push configuration, amplitude and location

Often, ARF amplitude and configuration are not precisely known. In addition, there might be an error in the estimated push location. In this section, we explore the effect of errors in push characteristics on the reconstructed results. We consider the irregular inclusion shape shown in figure 5(c). We keep all the inputs the same as in previous examples.

**Figure 7.**
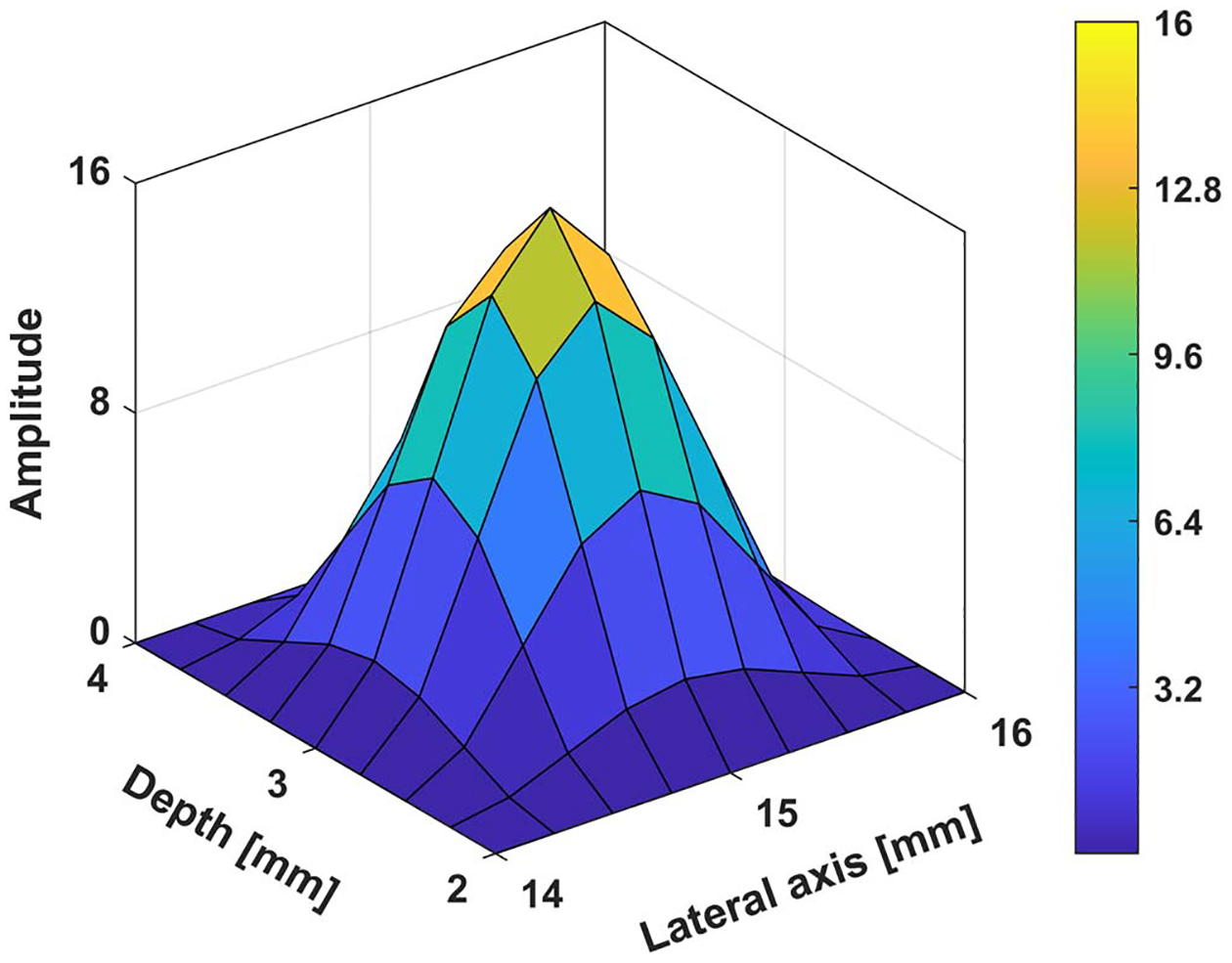
Modified ARF configuration.

Push signature and amplitude. To mimic error in push characteristics, we consider the modified configuration shown in figure 7, which is used for reconstruction (note that this is different in shape and amplitude compared to the default configuration in figure 1(b)). Note that in this example, we assume that the shape is known, and only the shear wave speed is to be reconstructed. This resulted in the correct reconstructed speed of $5.16{\text{ m s}}^{-1}$, which again indicates that the proposed approach is not very sensitive to push signature and amplitude.

##### Push location.

We inject 25% error in the lateral and vertical position of the ARF push. The reconstructed inclusion shear wave speed is $4.76{\text{ m s}}^{-1}$, with a relative error of −7.7%, indicating that the reconstruction is not overly sensitive to the push location.

##### Effect of combined errors.

We combine the errors in push configuration and location and add a 10 dB noise to the synthetic measurements. In addition, we use a circle to approximate the irregular inclusion. We conduct reconstruction with three separate realizations. The reconstructed inclusion shear wave speeds are 4.95, $5.3,5.11{\text{ m s}}^{-1}$, representing relative errors of −4,2.7, −0.97%, indicating that combining errors may have a cancellation effect. However, error combinations may also have additive effects and it would be best to minimize the input errors to the extent practical.

### 3.3. Homogeneous Inclusion with Unknown Background Modulus

**Figure 8.**
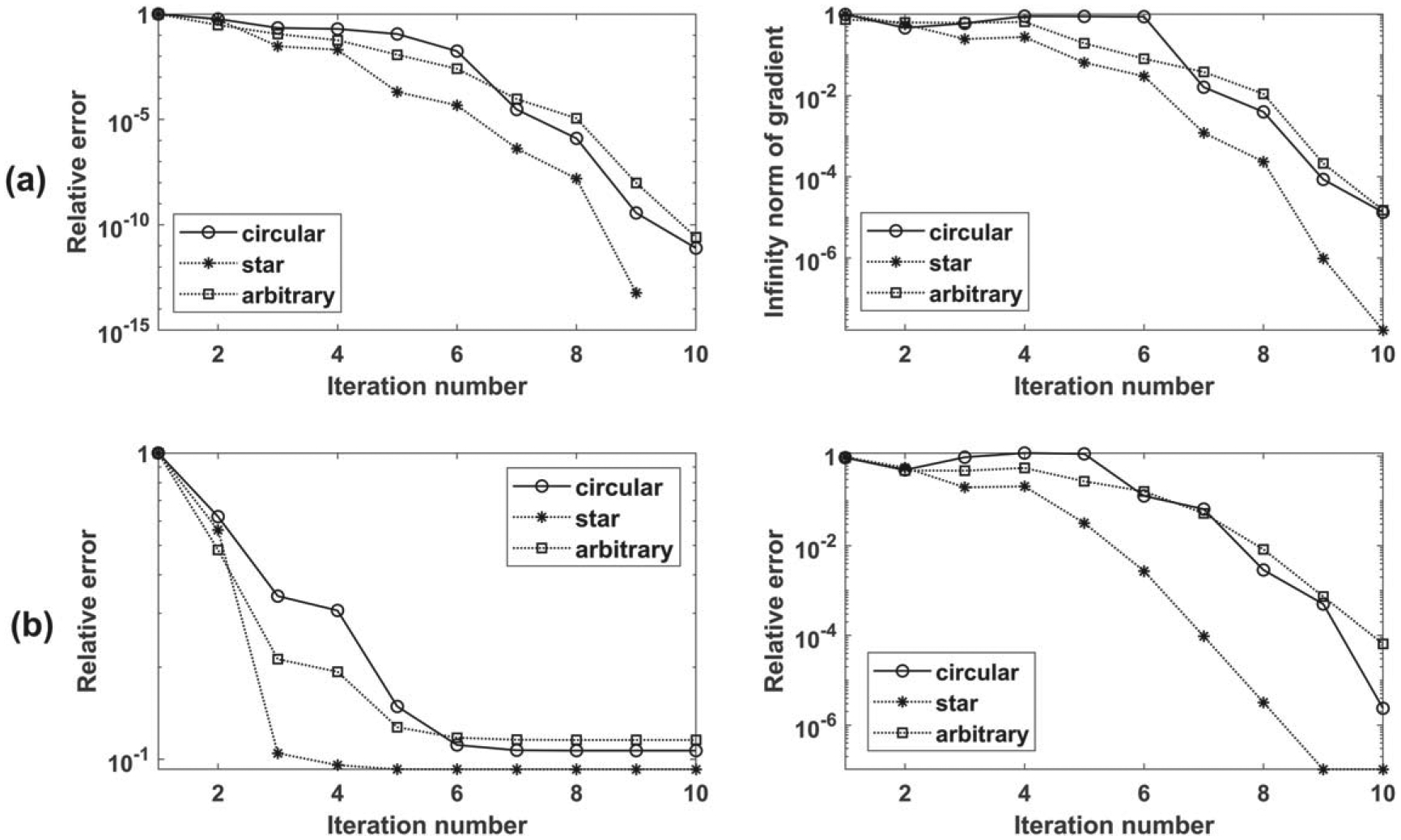
Convergence behavior of two-parameter inversion (a) without noise, (b) with 10 dB noise.

We now consider the case of unknown background shear wave velocity and attempt to simultaneously estimate both the background and inclusion shear wave velocities. We repeat the three tests in section 3.2.1 with the same inputs and setup except for three changes: (a) the initial velocity is assumed to be $2.0{\text{ m s}}^{-1}$ for both background and inclusion, (b) we invert for two parameters, namely the shear wave speeds of the inclusion and the background, (c) the default frequency range (i.e. 100 — 500HZ) is used with an increment of 100 HZ. The proposed approach is successful in reconstructing the true shear wave speed of the background and inclusion for all shapes. The convergence behavior for the three tests is shown in figure 8(a). To test the effect of noise, the reconstruction is repeated after adding 10 dB noise to the synthetic measurements. The results are summarized in table 2, and the convergence plot is shown in figure 8(b). The relative errors in reconstructed velocities are less than 2%, leading us to conclude that the proposed approach is capable of reconstructing both inclusion and background wave speeds accurately.

### 3.4. Heterogeneous inclusion

It is well-known that malignant tumors are often highly heterogeneous (Plodinec *et al* 2012). To facilitate the inversion of such heterogeneities, the ROI is split into small pixels, where each pixel has a unique shear wave speed, representing an unknown parameter in the inversion process. In what follows, we present two examples where the inversion is performed to reconstruct an array of parameters representing the shear wave speed distribution in the entire ROI, or a part of it.

#### Reconstruction without noise.

**Figure 9.**
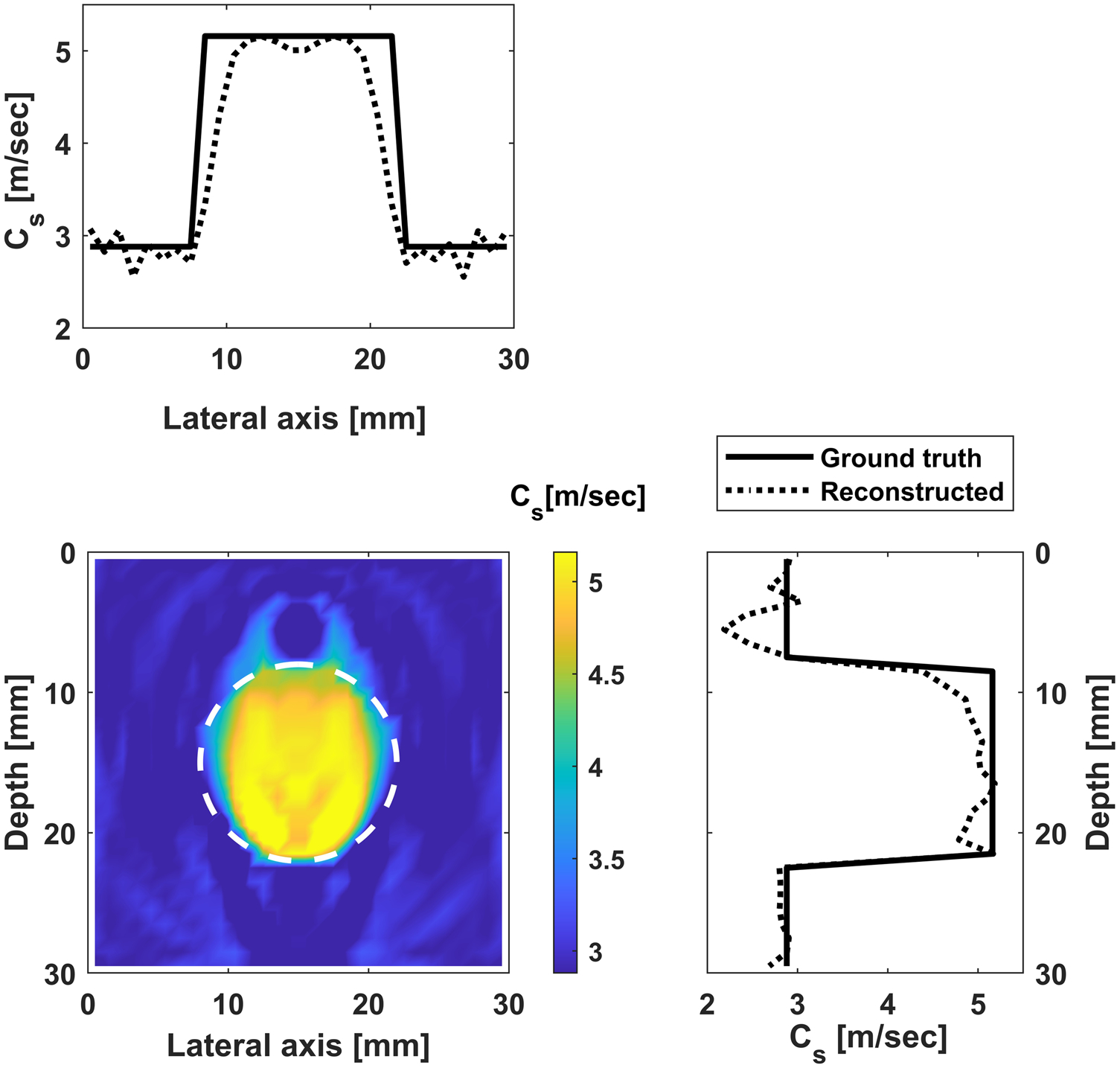
Reconstructed image for case (i): shear wave velocity distribution in the entire ROI. Bottom left is the contour of the shear wave velocity, while the top and side figures contain profiles along the horizontal and vertical diameters.

**Figure 10.**
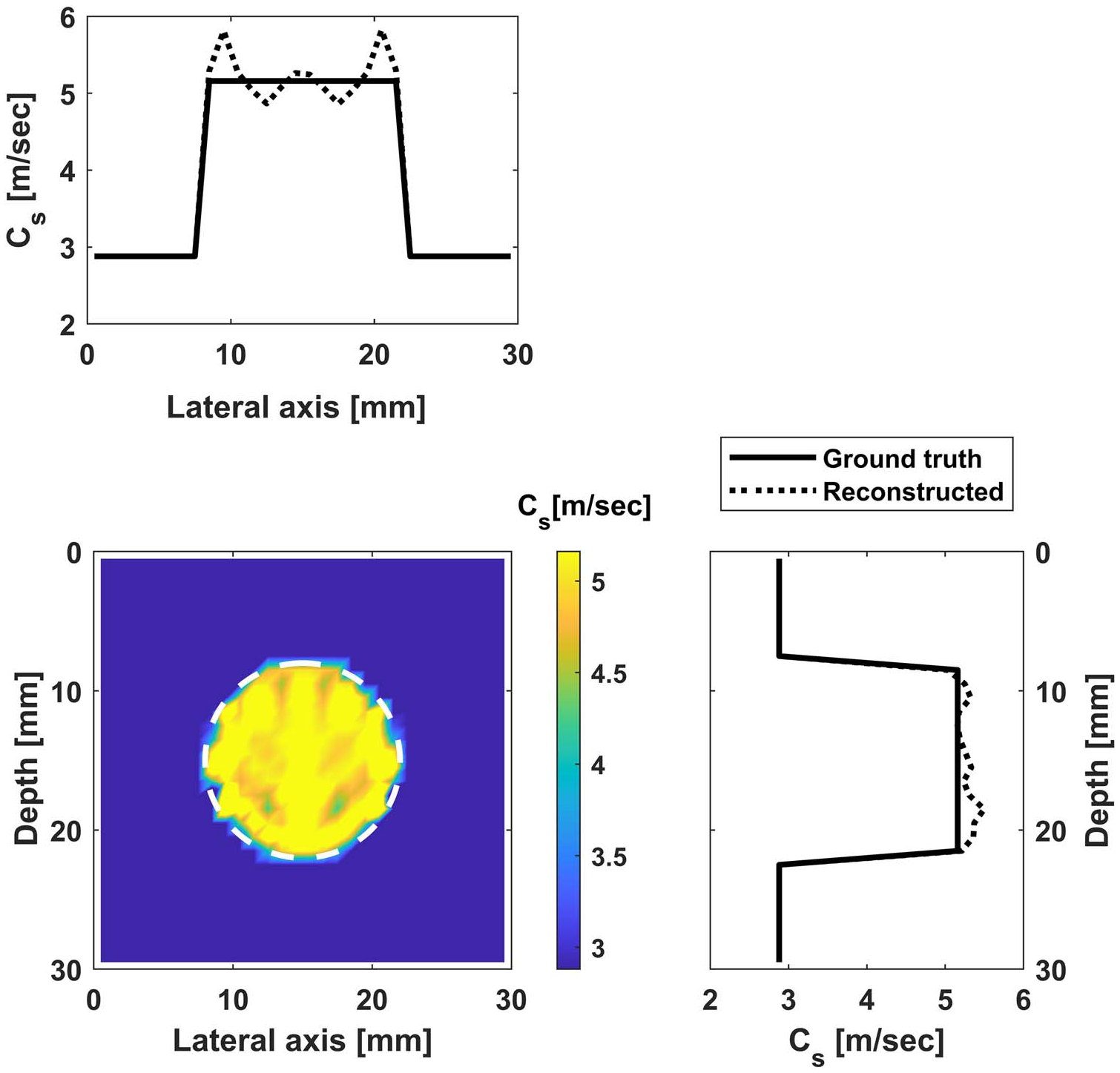
Reconstructed image for case (ii): shear wave velocity distribution inside the inclusion (with fixed background shear wave velocity). Bottom left is the contour of the shear wave velocity, while the top and side figures contain profiles along the horizontal and vertical diameters.

We test the performance of the proposed approach for two cases: (i) reconstruct all parameters in the ROI (900 parameters); (ii) limiting the parameters to be inverted inside a circle with a diameter of 14 mm (same as the inclusion diameter). The frequency range of 100–600 Hz with an increment of 10 Hz is used for inversion (more frequencies are used given that we are inverting for a much larger number of parameters, otherwise the inversion will be ill-posed). The measurements are considered on one single line with a total of 43 measurements. The initial values of the background and the inclusion are assumed to be $2.88{\text{ m s}}^{-1}$ and $3.6{\text{ m s}}^{-1}$; respectively. The reconstructed images are shown in figures 9 and 10, and the convergence behavior is shown in figure 13(a).

#### Reconstruction with noise.

**Figure 11.**
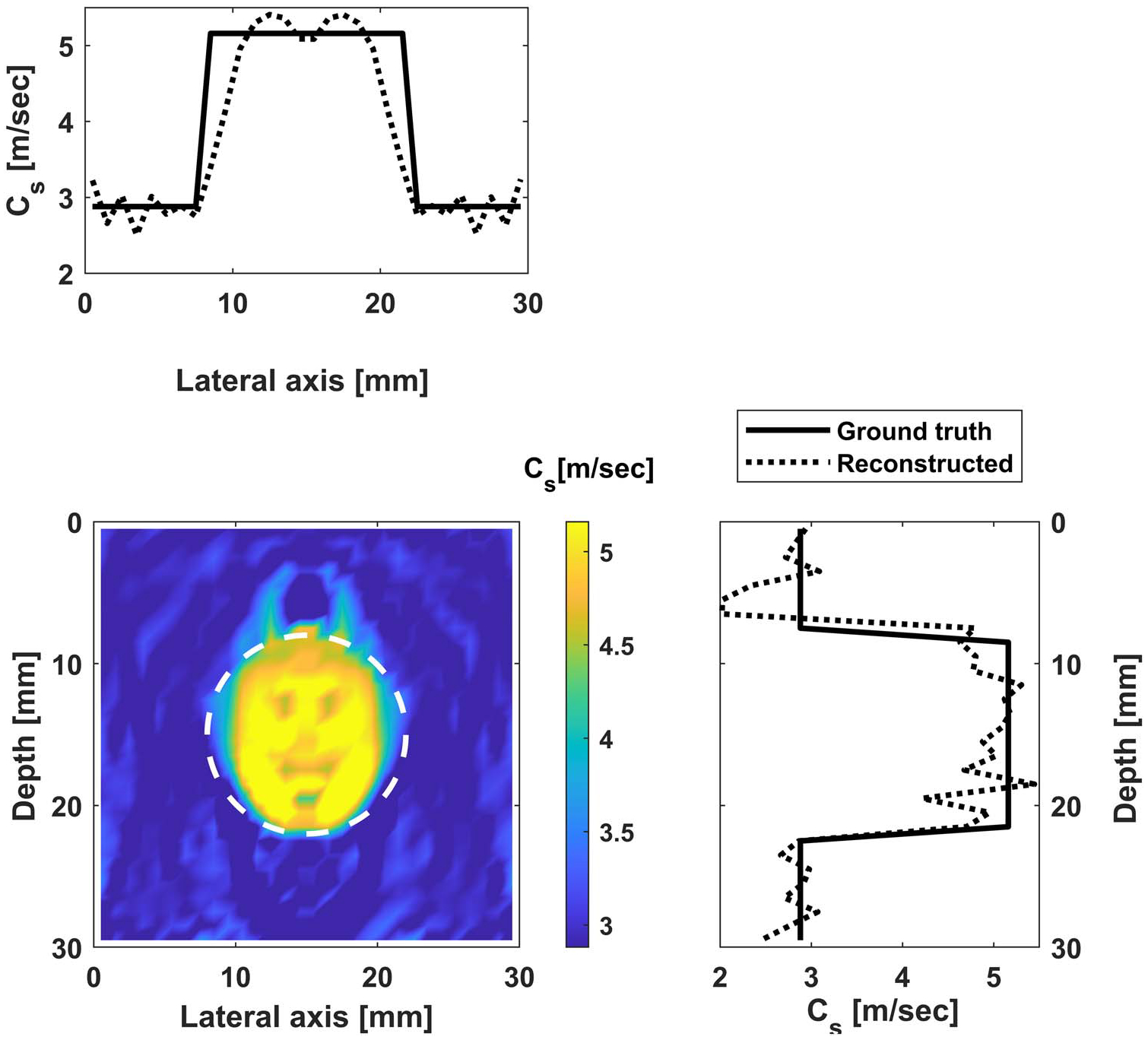
Reconstructed image for case (i) with noise: shear wave velocity distribution inside the inclusion (with fixed background shear wave velocity). Bottom left is the contour of the shear wave velocity, while the top and side figures contain profiles along the horizontal and vertical diameters.

**Figure 12.**
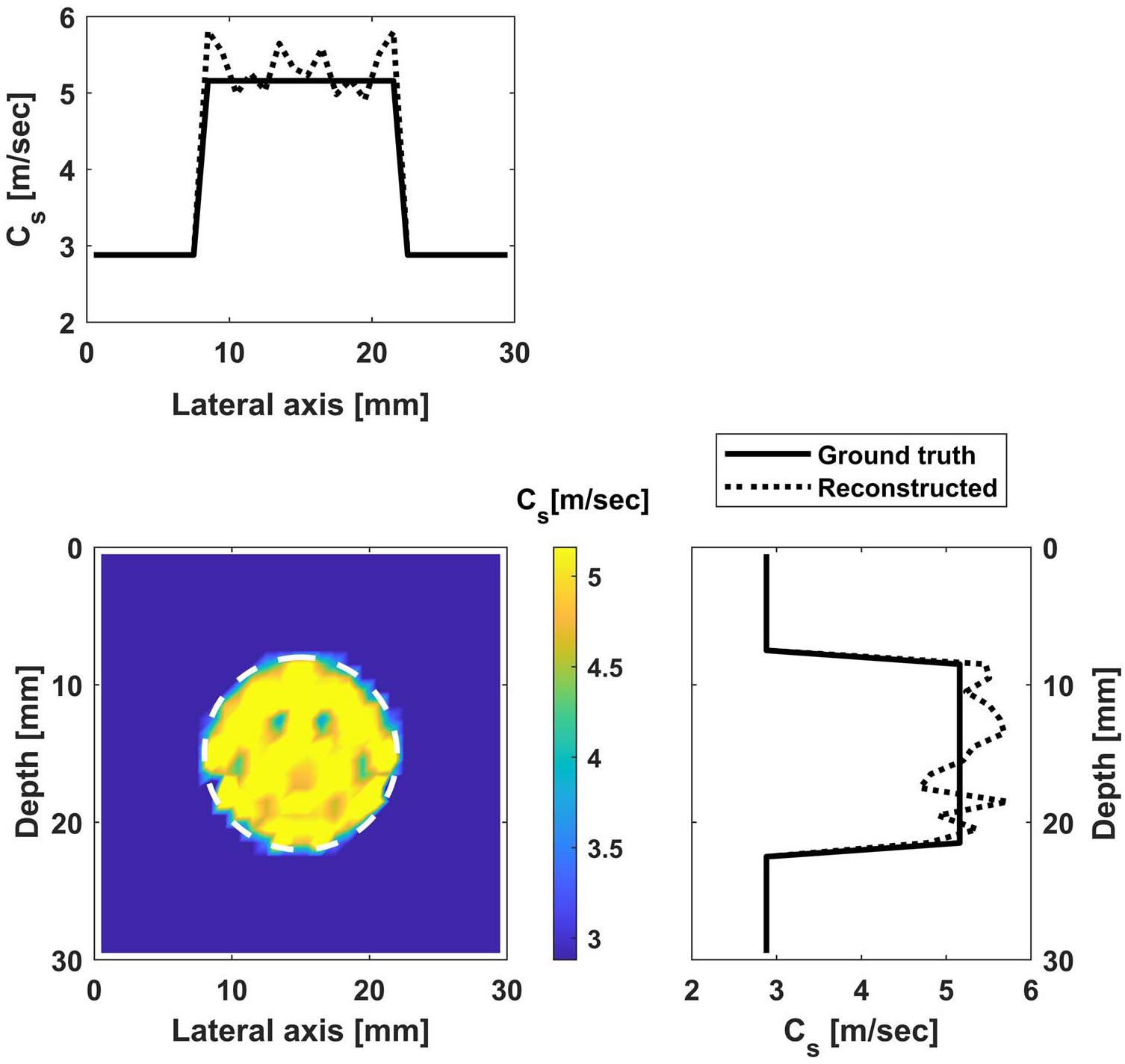
Reconstructed image for case (ii) with noise: shear wave velocity distribution inside the inclusion (with fixed background shear wave velocity). Bottom left is the contour of the shear wave velocity, while the top and side figures contain profiles along the horizontal and vertical diameters.

**Figure 13.**
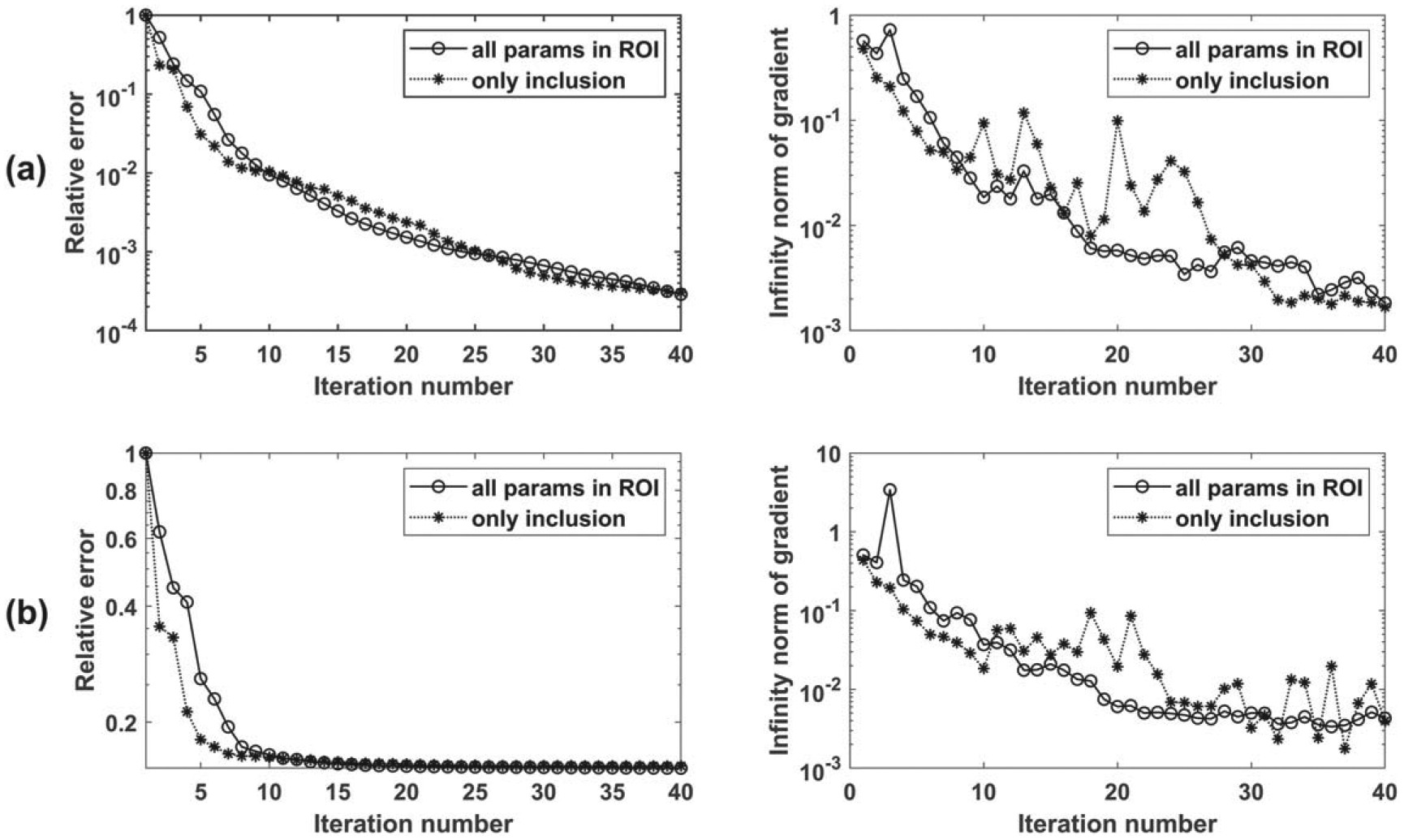
Convergence behavior for (i) inverting for the entire ROI and (ii) inverting for just the inclusion region. (a) without noise, (b) with noise. Left plots contain the cost functional and the right plots contain the norms of the gradient.

We repeat the reconstruction after adding 10 dB noise. The reconstruction results for cases (i) and (ii) are shown in figures 11 and 12. The convergence behavior is shown in figure 13(b). Comparing the reconstructed images with those without noise, we see more oscillations, especially in the vertical sectional profile for noisy data in both cases. The oscillations are more in the axial direction than in the lateral direction, which can be attributed to the measurements being on the vertical line, as explained using the underlying theory of adjoint-based gradient optimization, as follows. The misfit error is oscillatory due to noise and is concentrated on the vertical line as the measurements are restricted to the line. This misfit enters into equation (17) for the adjoint, which in turn enters in equation (16) for the gradient, and then for the image perturbation in equation (10). This process thus leads to oscillatory errors in the image, which are more along the vertical (measurement) line.

## 4. Discussion

We present a new reconstruction framework for ultrasound SWE which is based on three key ideas: first, full-waveform approach is used to better model the wave physics; second, a cross-correlation (CC) based cost functional is used to improve the convergence performance, especially with uncertain characteristics of the forcing function; third, the inversion is performed using measurements on a lower dimensional plane, i.e. use measurements on 1D line to reconstruct a 2D image. The first idea is aimed at mitigating the errors associated with plane wave and homogeneity assumptions of local reconstruction methods (conventional SWE). The second idea is based on the observation that CC functional is more convex than $L_{2}$ functional, leading to better convergence behavior. The motivation for the third idea is that conventional ultrasound transducer can only measure the wavefield in a plane while tumors to be imaged are 3D by nature; we attempt to mimic the setting in the simpler lower-dimensional settings where measurements on 1D line is used to reconstruct 2D images.

As discussed in section 2, CC functional emphasizes mainly phase information, as opposed to both phase and amplitudes that are highlighted by classical $L_{2}$ functionals. The CC functional is independent of the push information and is less sensitive to other errors, resulting in a much better behaving cost functional compared to the $L_{2}$ functional. This is illustrated in figure 6, where the new framework results in a wider radius of convergence compared to that when using the standard $L_{2}$ functional. Moreover, figure 6 indicates that the CC functional is more robust with respect to increasing the complexity of the inclusion shape. In section 3.2, when compared to the $L_{2}$ functional, the CC functional succeeded in reconstructing the shear wave speed in the inclusion, for all considered inclusion shapes and with noisy measurements.

The main motivation of inverting shear wave speed only in the inclusion is because it is assumed that the properties of the soft tissue surrounding the tumor are obtained by another modality. Notwithstanding this, we used synthetic experiments to reconstruct both background and inclusion shear wave speed, and the methodology is as effective as the estimation of the shear wave speed in the inclusion. Finally, we applied the proposed inversion to image heterogeneous tumors, leading to promising results, especially with known background properties.

Note that although all the measurements are synthetically generated, there are cases where there is not a complete match between the reconstructed and true values/images. This is attributed to the fact that we are solving an ill-posed nonlinear optimization problem with a gradient-based algorithm, thus, the reconstruction depends on many aspects such as the initial guess, parameterization, and the amount of data available. Therefore, we do not expect to have a complete match between the reconstructed shear wave speed distribution and the true values, unless we are inverting for just a handful of parameters, as done in sections 3.2 and 3.3.

A remark on computational cost is in order. For evaluating cost functional and the gradient, the proposed approach with CC functional requires almost the same computational effort as that using conventional $L_{2}$ functional. Additionally, given the improved behavior of the CC functional leading to faster convergence, the proposed approach is often faster than $L_{2}$ approach. With respect to the absolute computational cost, inversion for a single elastic modulus of the inclusion in section 3.2 took less than a minute on a desktop computer with Intel Xeon W2295 CPU and 128 GB RAM (note that the implementation is preliminary and no significant optimization is performed). For the most complex case of inverting for 900 parameters using 51 frequencies and 4761 degrees of freedom, it took about 10 min on the same computer. The run time may be significantly reduced using code optimization and utilizing the embarrassingly parallel nature of the forward model, where each frequency can reside on a separate processor.

The present work is based on a 2D antiplane shear approximation of the wave propagation in an elastic medium. In reality, tumors are 3D in nature, where the tissue can be considered incompressible. Thus, it is of importance to extend the framework to 3D where the objective would be reconstructing the 3D distribution of the shear wave speed. The main complexity here would be efficient forward modeling in 3D, considering incompressible elasticity. The ensuing mathematical formulation for adjoint-based gradient optimization will translate naturally from that presented in this work. However, given the increased complexity of wave propagation and scattering in 3D, it is not immediately clear if 3D inversion results would require further refinement of the cost functional, potentially needing regularization and/or re-parameterization.

Further complexities need to be considered when the method is finally extended to *in vivo* settings. Unlike the approximate version of ARF considered in this study, it would be beneficial to compute a more precise ARF excitation using acoustic simulation (see e.g. (Palmeri *et al* 2005, 2017)). In addition, while the proposed methodology shows clear promise for tumor imaging, it would be beneficial to extend the approach to diseases where the modulus has a diffused pattern over a large spatial range, e.g. liver fibrosis and steatosis (Angulo 2002, Rossman *et al* 2007). Furthermore, as discussed in the introduction section, inverting for viscoelasticity can provide an additional biomarker (Angulo 2002, Sinkus *et al* 2005a, 2005b, Rossman *et al* 2007), which is also a subject of future research.

Several additional approaches can be explored to enhance the performance of the proposed framework. These too are out of scope of the current work but discussed here for the sake of completeness. One such approach is to consider information from the other ultrasound modalities such as B-mode images and TOF results, to obtain initial estimates of shear wave distribution. Another refinement would be to consider multiple pushes as opposed to a single push used in this work; multiple pushes may enhance the quality of the images due to increased sensitivity to the modulus over a larger range. Multi-resolution reconstruction is another possible refinement, where the low-frequency content of the measurements would help in retrieving the coarse image of wave speed which can be then used as a starting point for higher frequency imaging, essentially helping expand the region of convergence. This strategy has been successfully tested in geophysics (see, e.g. (Bunks *et al* 1995, Eslaminia *et al* 2022, Eslaminia *et al* 2023)), and would likely help in our setting.

Reconstruction of tumors with sharp edges is of clinical importance. Researchers found a positive correlation between the sharpness of the edges and the grade of the tumor, and the propensity to metastasize (Lee *et al* 2016, Han *et al* 2020). In this work, we are interested in the medium frequency regime where the minimum wavelength is comparable to the size of the tumor itself. On the other hand, sharp edges reconstruction requires that there are sufficient wavelengths that are smaller than the smallest edge bumps. This in turn requires high-frequency waves; this too could be an area of future research, although there may be hardware limitations of ultrasound transducers.

## 5. Conclusion

We present a new cross-correlation based ultrasound SWE framework for imaging tumors. The method is tested using a synthetic experiment where 2D images are reconstructed using the wavefield measured on a single line. The proposed approach is shown to have several desirable features: (a) better modeling of wave physics, (b) partial measurements are sufficient for a good reconstruction, (c) the cost functional is less complex and has wider convexity and less nonlinearity, hence less sensitive to initial guess, (d) can handle heterogeneities, (e) is independent of the push amplitude, (f) is not very sensitive to errors in push location and configuration, (g) appears robust against measurement noise. Several *in silico* experiments with increasing complexity are presented, illustrating that the proposed framework is promising for imaging tumors. Extending the ideas to 3D elastic and viscoelastic inversion, and validation with phantom experiments are subjects of ongoing research.

## Figures and Tables

**Table 1.** Summary of the reconstruction results for different levels of noise.

SNR	True [${\text{m s}}^{-1}$]	Reconstructed [${\text{m s}}^{-1}$]	Percentage error
30 dB	5.16	5.14	−0.4
20 dB	5.16	5.184	+0.5
10 dB	5.16	5.186	+0.5

**Table 2.** Reconstructed shear wave speeds with 10 dB noise.

	Ground truth		Reconstructed		Percentage error	
Shape	Background [${\text{m s}}^{-1}$]	Inclusion [${\text{m s}}^{-1}$]	Background [${\text{m s}}^{-1}$]	Inclusion [${\text{m s}}^{-1}$]	Background	Inclusion
Circle	2.88	5.16	2.84	5.12	−1.4	−0.78
Star	2.88	5.16	2.89	5.25	+0.35	+ 1.94
Arbitrary	2.88	5.16	2.84	5.17	−1.4	+0.19

## Data Availability

The data cannot be made publicly available upon publication because they are not available in a format that is sufficiently accessible or reusable by other researchers. The data that support the findings of this study are available upon reasonable request from the authors.

## List of symbols

μ: Shear modulus
x: Position vector
ρ: Density
c: Shear wave speed
n: Unit normal
u: Particle displacement
𝒰: Particle displacement in frequency domain
$\ddot{u}$: Particle acceleration
f: Acoustic radiation force in time domain
ℱ: Acoustic radiation force in frequency domain
𝓐: Discretized wave operator
u: Discretized particle displacement
b: Discretized acoustic radiation force
m: Material parameter (shear wave velocity distribution)
$\overset{ˆ}{m}$: Optimum material parameter
$m_{0}$: Initial estimates of material parameter
𝒥: Cost functional (mismatch between measured and simulated responses)
d: Measured particle displacements (synthetic)
𝓟: Projection/restriction operator
T: Transpose
†: Conjugate transpose
v: Adjoint variable (vector)
$\tilde{v}$: Modified adjoint variable
g: Gradient vector
𝓗: Hessian operator (matrix)
ε: Tolerance
$\alpha_{k}$: Step size at iteration number k
α: Shifting factors
β: Scaling factors
∇: Gradient operator
∇.: Divergence operator
ℝ: Real number space
$\Omega_{ROI}$: Computational domain (region of interest, ROI)
Λ: Dirichlet-to-Neumann operator
Γ,∂Ω: Boundary of ROI
⊆: Subset of
Δ: Increment
∣ ∣: Absolute value of a scalar
∥ ∥: Norm of a vector
ℜ{}: Real part of a complex number
